# Rehydration modeling and characterization of dehydrated sweet corn

**DOI:** 10.1002/fsn3.3303

**Published:** 2023-03-15

**Authors:** Pratik Nayi, Navneet Kumar, Sagar Kachchadiya, Ho‐Hsien Chen, Punit Singh, Pratiksha Shrestha, Ravi Pandiselvam

**Affiliations:** ^1^ Department of Tropical Agriculture and International Cooperation National Pingtung University of Science and Technology Neipu, Pingtung Taiwan; ^2^ Department of Processing and Food Engineering, College of Agricultural Engineering and Technology Anand Agricultural University Godhra India; ^3^ Department of Food Science National Pingtung University of Science and Technology Pingtung Taiwan; ^4^ Institute of Engineering and Technology, Department of Mechanical Engineering GLA University Mathura Mathura India; ^5^ Department of Food Technology and Quality Control (DFTQC), Development Ministry of Agriculture and Livestock Development Kathmandu Nepal; ^6^ Division of Physiology, Biochemistry and Post‐Harvest Technology ICAR – Central Plantation Crops Research Institute (CPCRI) Kasaragod India

**Keywords:** mathematical modeling, pretreatment, rehydration characteristics, sweet corn

## Abstract

The increasing demand of rehydrated foods is due to its better storage stability at ambient conditions and not requiring refrigeration. Prior to drying at 55, 60, 65, and 70°C in a hot air tray dryer, hot water blanching (H_B_), steam blanching (S_B_), and microwave blanching (M_B_) were employed as pretreatments. Rehydration of dried pretreated sweet corn kernel was performed in boiling water. The pretreatments and drying temperatures were independent factors that affected the dependent factors such as rehydration ratio, total sugar, ascorbic acid, geometric mean diameter, color, sensory evaluation, water absorption, mass, and geometric mean diameter. Peleg, Weibull, and newly proposed models were considered to describe the change in moisture content during rehydration. The proposed model performed better than other models and indicated the rise in equilibrium moisture content of rehydrated sweet corn with an increase in dehydration temperature of sweet corn due to higher *R* ^2^ (0.994), and lower chi‐square (0.005) and RMSE (0.064). The rehydrated sweet corn obtained from samples processed with microwave blanching and dehydration at 70°C showed higher retention of total sugar, ascorbic acid, geometric mean diameter, and color.

## Graphical abstract

The sweet corn sample was blanched using different methods. The microwave blanching followed by dehydration (70°C drying temperature) resulted in good quality product with high sensory attributes.

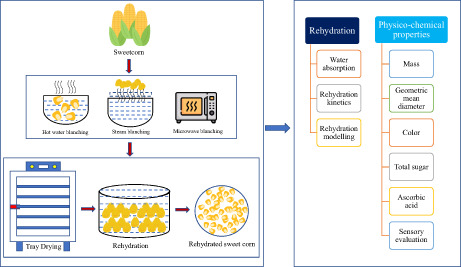

## 1 INTRODUCTION

Sweet corn (*Zea mays* L.) is one of the popular agricultural products used in different types of dishes. The storage life of sweet corn is very less, and its sweetness and tenderness are lost quickly. Pretreatment such as hot water blanching (H_B_), steam blanching (S_B_), and microwave blanching (M_B_) are popular and work on the principle of inactivation of the enzyme, which remains responsible for the faster deterioration of the produce (Pandiselvam et al., 2022). The drying time is also reduced by blanching and thus nutritional quality is also improved (Pravitha et al., 2022).

Generally, dehydrated food products need to be rehydrated before their edible use (Gan et al., 2016). The nutritional and sensory characteristics are affected by the process of dehydration followed by its rehydration (Zhu et al., 2022). The gain of moisture lost during drying is also dependent on the temperature of drying (Singh & Talukdar, 2020). A faster and shorter dehydration period enhances the absorption capacity of the dehydrated food material (Dehghannya et al., 2018).

A rehydration study is required for identifying the quantum of damage in the structure of food during drying, which overall affects the rehydration characteristics of foods (Liu et al., 2021). The swelling of the food material and imbibing of water into dehydrated food takes place during rehydration along with the leaching of soluble solutes from the food (Sagar et al., 2018; Singh et al., 2022). The quality always remains a major concern along with energy conservation for optimizing any dehydration process (Zhang et al., 2015). The structural variation in absorption behavior and structural matrix due to temperature change was reported by Mounir (2015), although the change in absorbed water remained nonsignificant.

Mass transfer kinetics modeling throughout the rehydration process can be described using empirical models. Fick's second law is used for preparing any diffusion models (İlter et al., 2018). The model proposed by Peleg is popular for various foods with porous structures due to its simplicity to other equations (Zielinska & Markowski, 2016). The Weibull model is also popular in food engineering applications due to its simplicity, which uses the probabilistic approach and also enhances the notable performance (Serment‐Moreno, 2021). Because of its versatility and simplicity, the standardized Weibull distribution model has recently been effectively employed to assess the drying kinetics of agricultural goods (Dai et al., 2019). The dehydrated sweet corn may remain a promising stable product, which can be used in several food applications like salads, pizza, sweet dishes, etc. after rehydration. However, no research has been carried out on the pretreatment such as hot water blanching (H_B_), steam blanching (S_B_), and microwave blanching (M_B_) for the determination of rehydration characteristics for rehydrated sweet corn. Therefore, the study was undertaken to observe the rehydration kinetics of dried sweet corn kernels with different pretreatments and drying temperatures to examine the probability of modeling of rehydration processing.

## 2 MATERIALS AND METHODS

### 2.1 Raw material

The raw material was procured from Godhra, Gujarat, India. Before drying experiments, the sweet corn kernels (Variety: Madhuri) were separated manually with a hand knife. The moisture content of the procured sweet corn kernel on a wet basis was 75 ± 3.3%.

### 2.2 Pretreatments

The sweet corn kernels were blanched using hot distilled water at 100°C for 120 s, blanched using steam for the 90s, and blanched using a microwave at 900 W power level for 60 s. The durations of different blanching methods were selected in the preliminary blanching study according to the appropriateness of blanching (Kachhadiya et al., 2018).

### 2.3 Drying experiment

The blanched sweet corn kernels were dried in a tray dryer (M/s Nova Instruments Pvt. Ltd., India). The kernels were spread over the trays in 5 mm thickness for drying in thin layers. The dryer consists of a heater for heating the air, which is blown using forced air convection. The tray was loaded with the sample amounting to 4.67 ± 0.1 kg/m^2^ as tray load. The mass of the samples before and after the drying was noted using a digital weighing balance (Shimadzu Corporation, Japan, Precision 0.01 g). The kernels were dried at 55, 60, 65, and 70°C drying temperatures. The drying process endpoint was observed till no reduction in mass was observed.

### 2.4 Rehydration experiment

Rehydration experiments were performed in boiling water in a beaker kept on a hot plate. At every five‐minute interval, the sweet corn kernels were taken out from the beaker. The samples were placed on tissue paper for removing surface moisture before weight measurement of the samples (Ranganna, 2000).

### 2.5 Rehydration kinetics modeling

The equilibrium moisture content cannot be estimated independently during rehydration due to many changes with long steeping times, therefore, becoming difficult to measure. The additional parameters for equilibrium moisture content in the rehydration kinetics models are used as suggested by Peleg's model (Patero & Augusto, 2015). Two already existing models namely Weibull's model and Peleg's models were used. Furthermore, a newly proposed model was also used, which was conceptualized based on the logarithmic variation considering the opposite trend of moisture content during rehydration in comparison to dehydration in thin‐layer drying models. All three models are represented in Table 1.

### 2.6 Rehydration ratio

The rehydration of the dehydrated sweet corn kernel was studied in terms of the rehydration ratio. It is defined as the ratio of the mass of rehydrated samples to the dried samples and can be represented by the following Equation 1. (Link et al., 2017).

(1) $$\text{Rehydration ratio}\;\left(\mathrm{RR}\right)=\frac{\text{Mass of rehydrated sample},g\;}{\text{Mass of dried sample},g}$$

### 2.7 Rehydration modeling

Rehydration needs a long steeping time and models used for rehydration modeling incorporate additional parameters for equilibrium moisture content (Lopez‐Quiroga et al., 2020). In this study, the experimental rehydration data of sweet corn kernel were converted to the moisture content at different treatments. The Weibull's, Peleg, and proposed new models were used to fit the moisture content with respect to the time for rehydration.

Weibull model can be expressed as:

(2) $$X_{\mathrm{wt}}=X_{\mathrm{eq}}+\left(X_{w0}-X_{\mathrm{eq}}\right)\exp\left[-{\left(\frac{t}{\beta }\right)}^{\alpha }\right]$$

where, X_wt_, X_wo_, and X_eq_ represent average instantaneous moisture content at any instantaneous time *t*, initial, and equilibrium moisture contents, respectively. The kinetic parameters are represented as *α* and *β* for this model. The behavior of the product during rehydration is indicated byα. The initial speed of rehydration increases with a decrease in αvalue and vice versa. The rehydration speed also has an inverse relation with the *β* parameter of the model (Lopez‐Quiroga et al., 2020).

Peleg's model can be expressed as:

(3) $$X_{\mathrm{wt}}=X_{w0}+\left[\frac{t}{k_{1}+\left(k_{2}\times t\right)}\right]$$

The Peleg rate constant is represented by the k_1_ parameter, Peleg capacity constant is represented by k_2_ related to equilibrium moisture content. At the time *t* = ∞, the equilibrium moisture content can be represented by:

(4) $$X_{\mathrm{eq}}=X_{w0}+\frac{1}{k_{2}}$$

The mechanism of rehydration indicates the absorption of water in the kernels, which is opposite to the mechanism of dehydration by the removal of moisture. Therefore, a new model is proposed based on opposite trends of the thin‐layer drying model, that is, logarithmic in place of exponential models, and is represented as:

(5) $$X_{\mathrm{wt}}=X_{\mathrm{eq}}+\left(X_{w0}-X_{\mathrm{eq}}\right)\ln\left(k*t\right)$$

Where k is a parameter indicating the rehydration rate.

The experimental data were analyzed statistically using OriginPro 9.4 (Origin Lab, Massachusetts). The models were evaluated to check the goodness of fit using *R* ^2^, root mean square error (RMSE, Equation 6), and chi‐square (*χ* ^2^, Equation 7). It is advisable to fit the model if the lower ancillary factors value and *R* ^2^ value is significant (Manikantan et al., 2022).

(6) $$\text{RMSE}=\sqrt{\frac{\sum_{i=1}^{N}{\left({\mathrm{MR}}_{\exp,i}-{\mathrm{MR}}_{\text{pred},i}\right)}^{2}}{N}}$$

(7) $$\chi 2=\frac{\sum_{i=1}^{N}{\left({\mathrm{MR}}_{\exp,i}-{\mathrm{MR}}_{\text{pred},i}\right)}^{2}}{N-Z}$$

### 2.8 Total sugar and ascorbic acid

The method was used for the sweetcorn samples after rehydration to determine the total sugar and the 2,6‐dichlorophenol‐indophenol method was used for the determination of ascorbic acid (Kumar et al., 2022).

### 2.9 Geometric mean diameter

The random selection of all three mutually normal dimensions of kernels was done and measured by a digital vernier caliper (M/S Mitutoyo Measuring Instruments Co. Ltd., China). The mentioned below expression by Desai et al. (2019) was used for the calculation of the geometric mean diameter (*D*_*g*_):

(8) $$D_{g}={\left(\mathrm{LWT}\right)}^{\frac{1}{3}}$$

where, L, W, and T are mutually normal dimensions and can be used to represent the length as the maximum dimension, width as normal to length, and thickness as normal to the length and width. All the dimensions were measured in millimeters (mm).

### 2.10 Total color difference

The color change was estimated using the following Equation (9). The subscript ‘₀’ refers to the color value of the fresh kernel. The total color difference should be as minimum as possible to maintain the color of the kernels and can be represented as:

(9) $$\mathrm{\Delta E}=\sqrt{{\left({L_{0}}^{*}-L^{*}\right)}^{2}+{\left({a_{0}}^{*}-a^{*}\right)}^{2}+{\left({b_{0}}^{*}-b^{*}\right)}^{2}}$$

where, L*, a*, and b* represent the degree of lightness to darkness with an initial value of Lₒ*, redness to greenness with an initial value of aₒ*, and yellowness to blueness with an initial value of bₒ*, respectively (Jeevarathinam et al., 2021; Pravitha et al., 2022).

### 2.11 Sensory evaluation

Sensory evaluation was conducted for rehydrated samples, which were kept in boiling water for 60 minutes (Xu et al., 2018). A semi‐trained panel of 15 panelists comprising faculty and postgraduate students were asked to access the rehydrated samples based on a hedonic rating test ranging from 1 to 9 indicating 1 as ‘dislike extremely’ and 9 as ‘like extremely’ according to their opinion for color, odor, taste, and overall acceptability (Ranganna, 2000). The samples were kept on a Petri dish and tests were conducted in uniform light, and the background of the coded samples was not informed to the panelists.

### 2.12 Statistical analysis

Rehydration characteristics and physicochemical parameters of rehydrated sweet corn were determined in triplicates. The statistical analysis was performed using Origin‐Pro9.2 (Origin Lab Corporation, Northampton, MA, USA) and Microsoft Excel (MS office 2016). For the mean comparison, 5% level of significance was set.

## 3 RESULTS AND DISCUSSION

### 3.1 Water absorption

**FIGURE 1:**
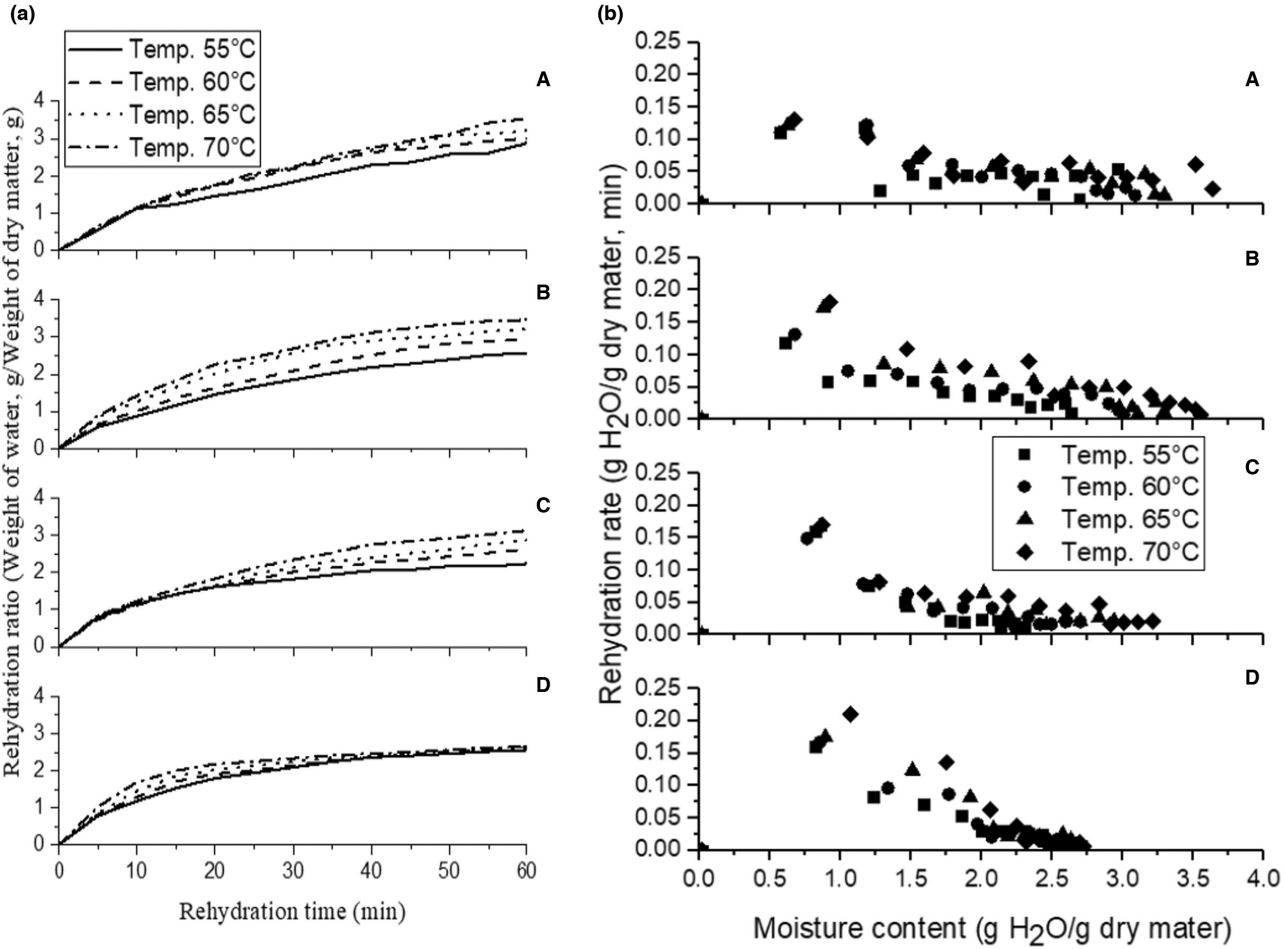
(I) Rehydration ratio and (II) rehydration rate during rehydration of sweet corn kernels at selected temperature with pretreatments (a, control; b, hot water blanched; c, steam blanched; d, microwave blanched).

The rehydration ratio of sweet corn samples dried before pretreatment and different temperatures is shown in Figure 1 (I). The rehydration ratio varied from 2.88 to 3.54, 2.56 to 3.46, 2.23 to 3.13, and 2.56 to 2.65 with control, H_B_, S_B_, and M_B_ samples for selected temperature ranges (55–70°C), respectively. A very small amount of water (0.2–0.3 g) is absorbed by the dehydrated sweet corn kernels. The increase in water uptake was observed with an increase in temperature. The collapse of starch granules and formation of colloidal solution due to the solubilization of amylopectin and amylose occurs due to an increase in temperature, thus an increase in water absorption with an increase in rehydration might be observed (Nadaf et al., 2021). The results are in line with the findings reported by other researchers for rehydration studies of several dried foods. Darvishi et al. (2015) observed a similar effect of temperature on the increase of rehydration ratio for dried soybean in fluidized bed drying. It was also found that increasing water temperature up to 70°C can reduce the rehydration time for the stink bean seed (Nisoa et al., 2021).

The cellular and structural disruption takes place during the drying of kernels, which affects the degree of rehydration of samples, which indicates the inability to rehydrate the product completely by imbibing the water (Miano et al., 2017). It can be further noted from (Figure 1I) that microwave‐blanched samples have retained the structures at all the drying temperatures and showed similar behavior of rehydration with respect to control, H_B_, and S_B_ samples. The rehydration ratios of dried microwave‐blanched (M_b_) samples were also less indicating limited absorption of water in the samples. It is also observed from (Figure 1I) that the rehydration ratio increased continuously till the rehydration time of 60 minutes in control and H_B_ samples, while it became consistent in the microwave in all the samples and some of the steam‐blanched samples.

The results in Figure 1 (II) indicate that rehydration rates were higher for all samples when the drying air temperature was increased. This is likely due to the fact that higher temperatures result in shorter drying times, leading to less structural and cellular damage. It was reported that minor disruption of cell wall polymers for orange by‐products during dehydration was about 50–60°C (Garau et al., 2007). Similar findings were observed in the study for purple‐fleshed sweet potatoes, where the compact tissue of the dried product was formed through continuous drying which resulting in a comfortable and steady destruction of the cells and structure (Liu et al., 2017).

### 3.2 Rehydration kinetics

The Peleg parameters k_1_, k_2_, and Xeq are shown in Table 1, which were obtained for control, H_B_, S_B_, and M_B_ samples and dried at 55–70°C. The Peleg model was fitted to the rehydration moisture content of the samples during the rehydration process. The estimated values of k_1_ ranged between 6.90 and 9.30 for control, 4.87–8.75 for H_B_, 4.9–6.2 for S_B_, and 2.6–5.2 for M_B_. The values of k_1_are in agreement with the values reported from 0.44 to 13.90 min‐kg dry matter/kg water for various foodstuffs at temperatures from 15 to 100°C (García‐Pascual et al., 2005). The constant k_1_ can be associated with the mass transfer rate and diffusion coefficient (Parlak, 2015). Similarly, the estimated values for k_2_ are varied between 0.146 and 0.208 for control, 0.196–0.234 for H_B_, 0.213–0.277 for SB, and 0.286–0.327 M_B_. The lower values of k_1_ being the rate constant indicates faster rehydration initially. The k_2_ represents the capacity constant, the lower value also affects the rehydration of samples (Johnny et al., 2015). Therefore, rehydration of microwave samples was more initially due to the lower value of k_1_, while rehydration was more in the case of control samples. It is interesting to note that k_2_ increased with an increase in temperature, indicating more value of capacity constant.

**TABLE 1:** Statistical results obtained from different models.

Drying temperature (°C)	Control						Hot water blanching						Steam blanching						Microwave blanching					
Peleg's model																								
	k1	k2	Xeq	*R* ^2^	ꭓ^2^	RMSE	k1	k2	Xeq	*R* ^2^	ꭓ^2^	RMSE	k1	k2	Xeq	*R* ^2^	ꭓ^2^	RMSE	k1	k2	Xeq	*R* ^2^	ꭓ^2^	RMSE
55	8.13	0.146	6.848	0.995	0.005	0.071	7.86	0.196	5.105	0.997	0.002	0.046	6.01	0.213	4.694	0.996	0.003	0.053	5.2	0.286	3.512	0.998	0.001	0.026
60	7.56	0.176	5.682	0.997	0.003	0.051	4.87	0.197	5.093	0.998	0.001	0.038	6.2	0.246	4.073	0.993	0.005	0.068	4.2	0.311	3.230	0.998	0.001	0.028
65	9.30	0.196	5.110	0.986	0.011	0.098	5.34	0.211	4.755	0.996	0.003	0.055	4.9	0.261	3.851	0.997	0.001	0.028	3.4	0.321	3.131	0.996	0.001	0.043
70	6.90	0.208	4.817	0.998	0.001	0.037	8.75	0.234	4.293	0.998	0.001	0.028	6.2	0.277	3.627	0.997	0.001	0.038	2.6	0.327	3.074	0.996	0.002	0.044

Weibull's model																								
	β	α	Xeq	*R* ^2^	ꭓ^2^	RMSE	β	α	Xeq	*R* ^2^	ꭓ^2^	RMSE	β	α	Xeq	*R* ^2^	ꭓ^2^	RMSE	β	α	Xeq	*R* ^2^	ꭓ^2^	RMSE
55	53.68	0.685	5.160	0.998	0.002	0.041	43.45	0.853	4.205	0.998	0.002	0.039	39.86	0.765	4.333	0.998	0.001	0.035	23.18	0.780	3.075	0.999	0.001	0.021
60	53.43	0.824	5.011	0.998	0.002	0.042	40.45	0.862	4.125	0.999	0.001	0.033	82.85	0.632	4.255	0.998	0.001	0.035	15.80	0.839	2.737	0.998	0.001	0.030
65	48.78	0.599	4.010	0.993	0.005	0.065	25.97	0.919	3.673	0.997	0.002	0.046	37.95	0.710	3.570	0.999	0.001	0.016	10.42	0.820	2.701	0.997	0.003	0.048
70	31.22	0.926	3.694	0.998	0.002	0.040	23.90	0.852	3.526	0.999	0.001	0.022	20.26	0.700	3.401	0.999	0.001	0.018	12.54	0.891	2.645	0.995	0.002	0.039

Proposed Reverse of Lewis's model																				
	k	Xeq	*R* ^2^	ꭓ^2^	RMSE	k	Xeq	*R* ^2^	ꭓ^2^	RMSE	k	Xeq	*R* ^2^	ꭓ^2^	RMSE	k	Xeq	*R* ^2^	ꭓ^2^	RMSE
55	0.004	3.385	0.984	0.013	0.106	0.034	3.000	0.998	0.002	0.038	0.069	2.256	0.989	0.005	0.066	0.057	2.727	0.994	0.004	0.058
60	0.002	3.476	0.998	0.002	0.044	0.033	3.533	0.997	0.003	0.053	0.049	2.745	0.992	0.006	0.068	0.070	2.601	0.996	0.003	0.050
65	0.002	3.890	0.996	0.005	0.063	0.046	3.514	0.997	0.003	0.052	0.044	3.040	0.987	0.010	0.094	0.083	2.587	0.996	0.003	0.047
70	0.002	4.486	0.993	0.010	0.091	0.046	3.783	0.997	0.004	0.055	0.042	3.435	0.994	0.006	0.072	0.101	2.617	0.993	0.005	0.064

The Weibull's parameter α represents the shape factor, which varied between 0.599 and 0.926 for control, 0.853–0.919 for H_B_, 0.632–0.765 for S_B_ and 0.780–0.891 for M_B_ indicating wider variation of shape factor in control and minimum in H_B_ samples. Furthermore, the higher values of *α* indicate lower water absorption rates at the beginning of rehydration. That higher range of α was minimum for steam‐blanched sample indicates higher water absorption rate. The value of *α* varied between the already prescribed limit for foodstuffs between 0.2 and 1.0 (García‐Pascual et al., 2005). Another parameter *β* represents the rate of rehydration and the time required to achieve a 63% level of rehydration, which varied from 31.22 to 53.68 for control, 23.90–43.45 for H_B_, 20.26–82.85 for S_B_, and 10.42–23.18 in M_B_ sweet corn kernels, indicating minimum time is required for 63% of rehydration in microwave‐blanched samples followed by hot water‐blanched samples. The effect *β* on temperature also showed decreasing trend with increase in temperature. A similar variation was also observed in control and hot water‐blanched samples. The *α* parameters of the model do not get any linear influence by the drying temperature, while the parameter *β* showed a decreasing trend with the increase in temperature for control and hot water‐blanched samples. A similar trend for Weibull's parameters was also observed by Cox et al. (2012).

The newly proposed model parameter k varied from 0.002 to 0.004 for control, 0.033–0.046 for H_B_, 0.042–0.069 for SB, and 0.057–0.101 for M_B_ samples, which may be related to other parameters of Peleg and Weibull's model. The lower value of k represents slower rehydration in the case of control and a higher value of k represents faster rehydration of samples. This effect can be attributed to the collapse of the structure at longer blanching and drying operations, which may have caused lower diffusion coefficients.

**FIGURE 2:**
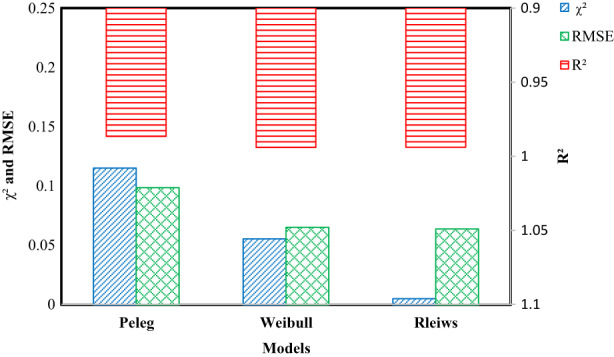
Performance of models for rehydration characteristics.

**FIGURE 3:**
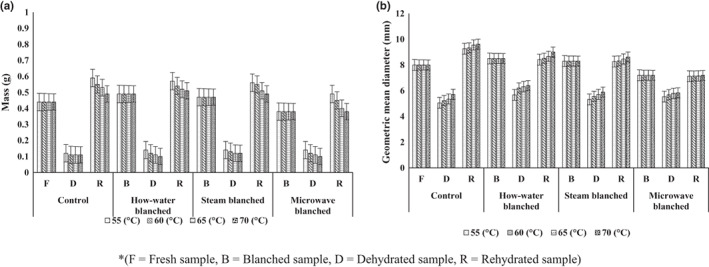
(I) Mass and (II) geometric mean diameter variation during dehydration and rehydration at different pretreatments and drying temperatures.

Table 1 shows the values of Xeq identified from the Peleg; Weibull model decreased with the increase in the temperature for every treatment. However, the equilibrium moisture content increased with the increase in drying temperature in the proposed model, which can be very well justified by Figure 1, which indicates more final moisture content of rehydrated sweet corn samples for the samples dried at higher temperatures. It can be observed from Figure 3 that the average *R* ^2^ values for Peleg, Weibull, and the newly proposed model were 0.0986, 0.994, and 0.994, respectively, whereas *χ* ^2^ and RMSE values were 0.115 and 0.987; 0.056 and 0.065; and 0.005 and 0.064 indicates that all methods fitted well to the rehydration data. However, the newly proposed model is recommended to have higher *R* ^2^ and lower *χ* ^2^ and RMSE values along with the advantage of using only one parameter indicating less complexity of the model (Figure 2).

### 3.3 Mass

The fresh kernel mass was about 0.44 g in fresh sweet corn. The mass of pretreated sweet corn kernels reduced from 0.11 to 0.14 g at drying temperatures from 55 to 70°C (Figure 3I). The mass of fresh samples was 0.44 g, which changed to 0.49, 0.47, and 0.38 g in H_B_, S_B_ and M_B_ of samples. The mass of control, H_B_, S_B_, and M_B_ samples were reduced to 0.11, 0.10, 0.12, and 0.10 g, respectively, which regained to 0.49–0.59, 0.51–0.57, 0.49–0.56, and 0.38–0.49 g, respectively. It can be noted from the graph that the sweet corn samples absorbed more moisture than fresh samples and over swelled in the case of control, H_B_, and S_B_ samples. The mass of samples after rehydration also increased with an increase in drying temperatures in all cases was also significant (*p* < .05). The lesser water absorbance of water was observed in microwave‐blanched (M_b_) samples and at a higher temperature, indicating an absence of over‐swelling of sweet corn kernels. The over‐swelling in control, H_B_, and S_B_ samples may be attributed to the occurrence of rupturing the texture of sweet corn during blanching followed by drying operations.

### 3.4 Geometric mean diameter

Figure 3(II) shows the effect of pretreatments (H_B_, S_B_, and M_B_) and drying temperature (55, 60, 65, 70°C) on the rehydration of sweet corn geometric mean diameter. It was observed that the geometric mean diameter increased in the case of H_B_ and S_B_, whereas it decreased in the case of microwave blanching. A minimum decrease in dimension was also observed after dehydration in microwave‐blanched samples, which is evident due to the minimum change in the structure of blanched sweet corn. The gain in dimension was more in all the rehydrated samples in comparison to fresh samples except microwave‐blanched samples, which may be due to the partial rupture of sweet corn internal structure in other blanching methods. The results are in line with the previous finding of an increase in the mass of samples. In the previous study, it was observed that due to gaining moisture the dimensions were augmented and it was also reported that deflation in the dimensions of sweet corn was due to losing the moisture during the microwave heating process (Kachhadiya et al., 2018). Similar results were also obtained by Popaliya and Kumar (2022) for sweet corn kernels by using microwave blanching and a reduction in the geometric mean diameter and mass of the sweet corn kernels sample was reported.

### 3.5 Total color difference

**FIGURE 4:**
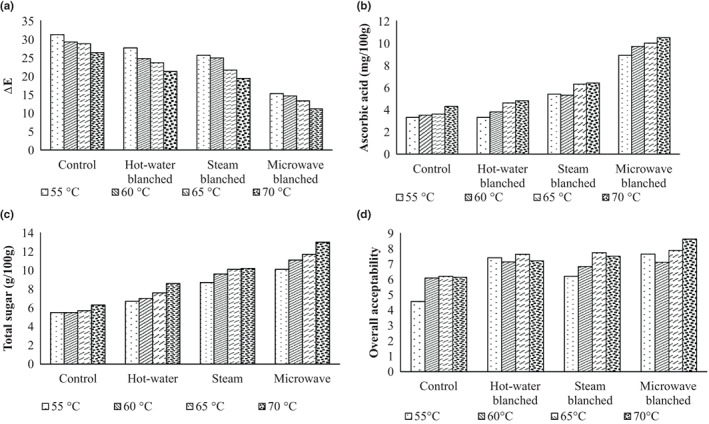
Variation of (I) total color difference, (II) ascorbic acid, (III) total sugar, and (IV) overall acceptability of sample after rehydration at different pretreatments and drying temperatures.

The total color difference after rehydration of sweet corn kernel of control, H_B_, S_B,_ and M_B_ samples with fresh samples was 31.23–26.34, 27.63–21.23, 25.61–19.36, and 15.23–11.12 at drying temperature varying between 55 and 70°C, indicating more retention color of microwave‐blanched samples (Figure 4I). The minimum change in color of microwave‐blanched samples during drying and rehydration is due to effective blanching and less destruction of color pigments. Retention of color L was found highest in the microwave‐blanched sample of purple flesh sweet potato. Microwave‐blanched treated samples have found a brighter appearance as compared to steam‐blanched samples. It was also reported that hot water‐blanched samples have worse color uniformity than microwave‐blanched samples (Liu et al., 2015). Delfiya et al. (2018) studied the microwave blanching effect and brine solution on the carrots and reported that the complete inactivation of enzymes causes color changes and also caused a decrease in total color. It was also reported that microwave blanching is more effective for the inactivation of enzymes and retention of total color values. The increase in total color changes due to the lower microwave power level and the reduction in total color changes just because of the longer time of microwave exposure (Srinivas et al., 2020).

### 3.6 Total sugar and ascorbic acid

Total sugar after dehydration of sweet corn kernel of control, H_B_, S_B_, and M_B_ samples varied from 5.21 to 5.53 g/100 g, 6.13 to 6.63 g/100 g, 6.61 to 7.26 g/100 g, and 7.21 to 7.41 g/100 g at drying temperature from 55 to 70°C indicating retention of higher sugar in microwave‐blanched samples (Figure 4(II)). The ascorbic acid after dehydration of sweet corn kernel of control, H_B_, S_B_, and M_B_ samples were 4.24–4.61 mg/100 g, 5.23–5.51 mg/100 g, 5.46–5.63 mg/100 g, and 5.83–6.01 mg/100 g, respectively (Figure 4(III)), at drying temperature varying between 55 and 70°C indicating higher retention of ascorbic acid in microwave‐blanched samples too, which is evident due to retention of vitamins and sugar in microwave‐blanched samples. Similar higher retention of ascorbic acid and total sugar of sweet corn was observed in microwave blanching in comparison to hot water and steam blanching (Kachhadiya et al., 2018).

### 3.7 Sensory evaluation

An increasing trend of overall acceptability with an increase in drying temperature was observed during the sensory evaluation of the rehydrated samples. The maximum sensory score of rehydrated samples was 8.62, indicating the highest preference for samples blanched with microwave and dehydrated at 70°C among the treatments in the study (Figure 4(IV)). The lower score at lower temperature drying may be attributed to the longer drying periods, while the lower score in sensory analysis in control, H_B_, and S_B_ samples may be attributed to the partial rupturing of kernels and color degradation of the kernels.

### 3.8 Compound correlation

This correlation describes the results of the study investigating the effect of different blanching methods and drying temperatures on the physical, chemical, and sensory properties of sweet corn. Compound correlation was used to analyze the data, which is a statistical technique used to identify the similarities and differences between multiple process variables and characteristics of the product.

**FIGURE 5:**
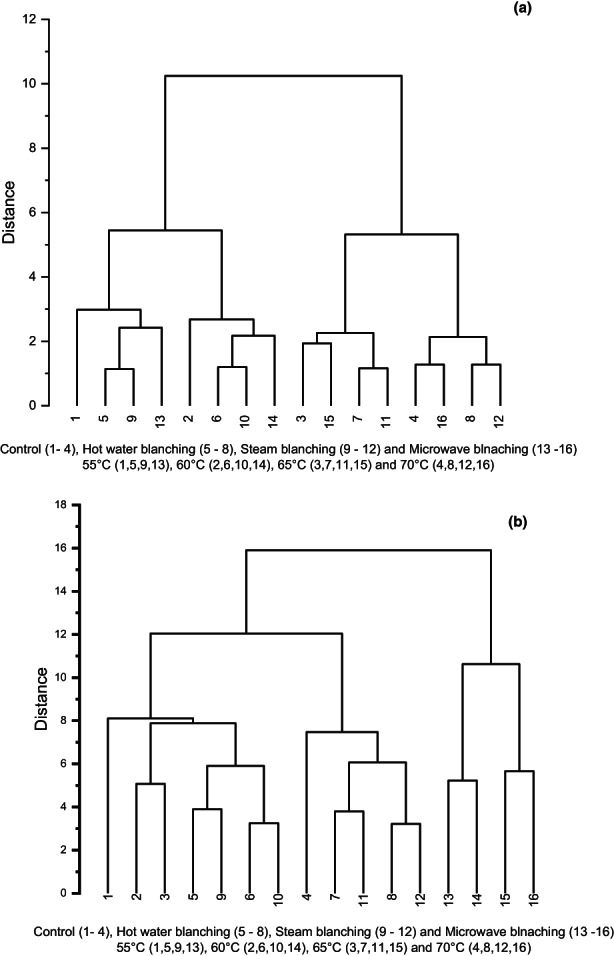
Compound correlation for (a) physical properties and (b) overall properties of rehydrated sweet corn.

In Figure 5a, a dendrogram was used to represent the compound correlation among all the treatments based on physical properties. The dendrogram shows that samples dried at 55 and 60°C are similar and can be kept in one cluster, while samples dried at 65 and 70°C form another cluster at about 50% of the total distance on the vertical axis, indicating the classification of both clusters based on temperatures. The subclusters of samples within each cluster based on drying temperature also suggest that the physical properties of samples dried at a specific temperature are more similar to each other than to samples dried at a different temperature. This indicates that the drying temperature has a more prominent effect on physical properties compared to the type of blanching methods used. The vertical distance of clusters reduced with an increase in temperatures, indicating that samples treated at higher drying temperatures had more similar physical properties. In Figure 5b, the overall compound correlation of physical, chemical, and sensory properties was analyzed. The results showed that microwave‐blanching (M_b_) samples had overall similar properties. The microwave‐blanching samples of 60 s dried at 70°C showed the highest overall acceptability and had higher retention of ascorbic acid and total sugar. The results also indicate that the type of blanching method and drying temperature significantly affect the overall properties of sweet corn.

Overall, the compound correlation analysis helped to identify the similarities and differences between multiple variables and allowed the researchers to draw conclusions about the effect of blanching method and drying temperature on the physical, chemical, and sensory properties of sweet corn.

## 4 CONCLUSION

The rehydration ratio varied from 2.56 to 3.54for all the samples dehydrated at the temperature range (55–70°C), indicating good rehydration of sweet corn samples. The average rehydration rates of sweet corn were 0.040–0.056 g water/g dry matter‐min. The newly proposed model is recommended to have higher *R* ^2^ and lower *χ* ^2^ and RMSE values along with less complexity of the model. Minimum changes in mass, geometric mean diameter, and color were observed in the case of microwave‐blanched samples. The total sugar of rehydrated samples varied from 5.21 to 7.41 g/100 g and ascorbic acid content varied from 4.24 to 6.01 mg/100 g. The highest sensory score of rehydrated samples was 8.62 for microwave‐blanched samples at 900 W power levels for 60 s and dehydrated at 70°C, indicating the suitability of rehydration of dehydrated sweet corn. Some recent studies highlight the importance of optimizing rehydration methods for sweet corn and other dehydrated vegetables, and the potential benefits of using novel processing techniques to improve the rehydration properties and overall quality of the final product. According to recent studies many researchers have reported that soaking in hot water at 50°C for 20 min was the most effective rehydration method for producing high‐quality, rehydrated sweet corn with good color, texture, and sensory properties. These findings can contribute to the development of effective strategies for preserving and utilizing surplus sweet corn, thereby reducing food waste and increasing food availability, particularly in areas with limited access to fresh produce.

## CONFLICT OF INTEREST STATEMENT

The authors declare that they have no conflict of interest.

## ETHICAL APPROVAL

This study does not involve human or animal experiments.

## Data Availability

Data will be made available upon request.
